# Does the intestinal microbial community of Korean Crohn’s disease patients differ from that of western patients?

**DOI:** 10.1186/s12876-016-0437-0

**Published:** 2016-02-29

**Authors:** Chang Soo Eun, Min-Jung Kwak, Dong Soo Han, A. Reum Lee, Dong Il Park, Suk-Kyun Yang, Yong Seok Kim, Jihyun F. Kim

**Affiliations:** Department of Internal Medicine, Hanyang University Guri Hospital, Guri, Korea; Department of Systems Biology and Division of Life Science, Yonsei University, Seoul, Korea; Department of Internal Medicine, Sungkyunkwan University, Seoul, Korea; Department of Internal Medicine, Ulsan University, Seoul, Korea; Department of Biochemistry, Hanyang University, Seoul, Korea

**Keywords:** Crohn’s disease, Intestinal bacteria, Korean, Pyrosequencing, Dysbiosis

## Abstract

**Background:**

Intestinal microbiota play an important role in maintaining the homeostasis of the host immune system. To analyze the alteration of the intestinal microbial community structure in Korean Crohn’s disease (CD) patients, we performed a comparative metagenomic analysis between healthy people and CD patients using fecal samples and mucosal tissues of ileocecal valve.

**Methods:**

16S rRNA genes from fecal samples or mucosal tissues of 35 CD patients and 15 healthy controls (HC) were amplified using a universal primer set and sequenced with GS FLX Titanium. The microbial composition and diversity of each sample were analyzed with the mothur pipeline, and the association between microbial community and clinical characteristics of the patients were investigated.

**Results:**

The contribution of bacterial groups to the intestinal microbial composition differed between CD and HC, especially in fecal samples. Global structure and individual bacterial abundance of intestinal microbial community were different between feces and ileocecal tissues in HC. In CD patients with active stage, relative abundances of *Gammaproteobacteria* and *Fusobacteria* were higher in both fecal and mucosal tissue samples. Moreover, the intestinal microbial community structure was altered by anti-tumor necrosis factor (anti-TNF) treatment.

**Conclusions:**

Our 16S rRNA sequence data demonstrate intestinal dysbiosis at the community level in Korean CD patients, which is similar to alterations of the intestinal microbial community seen in the western counterparts. Clinical disease activity and anti-TNF treatment might affect the intestinal microbial community structure in CD patients.

**Electronic supplementary material:**

The online version of this article (doi:10.1186/s12876-016-0437-0) contains supplementary material, which is available to authorized users.

## Background

Microbes in the body are tenfold more than the number of human cells, and encode 100 times more unique genes than human genomes. Intestinal microbiota are in close contact with mucosal immune system, affecting host physiology and maintaining homeostasis [1–3]. The incidence of Crohn’s disease (CD) has been reported to be increasing around the world, and while it is responsible for inducing chronic intestinal inflammation in a genetically susceptible host, the underlying mechanism is thought to result from an inappropriate and ongoing activation of the mucosal immune system driven by several stimuli such as intestinal microbiota and various environmental factors [4].

CD is relatively more prevalent in western population, and numerous genetic susceptibility loci have been identified [5–7]. However, well-known CD susceptible genes such as *NOD2* and *ATG16L1* did not show replication results in Asian population, suggesting a different genetic background [8, 9]. Although the genetic susceptibility loci differ between Asian and western population, the incidence of CD in Asian population is increasing with similar immunologic phenomena [10]. Therefore, it can be presumed that environmental factors, especially intestinal commensal bacteria, besides genetic factors, play a fundamental role in the development of CD. In fact, it is well known that the intestinal microbial community of western CD patients demonstrates dysbiosis different from healthy population [11, 12]. However, there is no published study demonstrating intestinal microbial profiles of Korean CD patients using high throughput sequencing methods.

In the present study, we examined and compared the fecal and mucosal microbial community of Korean CD patients and healthy controls (HC) by applying a next-generation sequencing method after isolation of microbial DNA.

## Methods

### Study population

We collected stool or mucosal tissue specimens from CD patients who underwent colonoscopic examination and from similar age group of HC who also underwent colonoscopic examination for screening in Hanyang University Guri Hospital. Controls consisted of healthy subjects aged 18 years and older. HC had no evidence of active inflammatory conditions of the gastrointestinal tract. Patients with a history of inflammatory bowel disease, colon cancer, colonic resection, or hospital admission in the previous 3 months, or presence of chronic illness (such as renal failure, diabetes, or cardiopulmonary diseases), were excluded from HC. All of the enrolled CD patients and HC had not taken antibiotics in the previous 3 months. The study was approved by the institutional review board of Hanyang University Guri Hospital (GURI 2012-05-022). Written informed consent for participation and publication was obtained from all participants prior to the enrollment of this study.

### Sample collection and sequencing

Stool samples were collected in sterile containers at home before the start of bowel preparation and stored at 4 °C. Upon arrival at the hospital, the stool samples were frozen at −80 °C. Mucosal tissue samples were taken from the ileoceal valve area using sterile endoscopic biopsy forceps during colonoscopic examination and immediately stored at −80 °C. After homogenization, DNA was extracted using a phenol/chloroform extraction method combined with physical disruption of bacterial cells and the UltraClean microbial DNA Isolation kit (Mo Bio Laboratories, Carlsbad, CA, USA). The DNA concentration and quality were determined by agarose gel electrophoresis (1 % wt/vol agarose in Tris-acetate-EDTA buffer) and with a NanoDrop ND-1000 spectrophotometer (NanoDrop Technologies, Wilmington, DE, USA). DNA spanning the V1-V3 region of bacterial 16S rDNA was amplified using a barcoded universal primer (8F 5′-barcode sequence-linker sequence (AC)-GAGTTTGATCMTGGCTCAG-3′ or GGGTTCGATTCTGGCTCAG for *Bifidobacterium*) and 518R 5′-barcode sequence-linker (AC)- WTTACCGCGGCTGT GG-3′). Sequencing was conducted with Roche GS FLX Titanium platform according to the manufacturer’s specifications (DNA Link Inc., Republic of Korea).

### Data analysis

#### Read trimming

The sequences were basically analyzed using the MOTHUR pipeline [13]. Sequence trimming was conducted in sixth steps. In the first step, the sequence trimming was conducted at the flowgram level with recommended values for Titanium data that were minimum flow value of 360 and maximum flow value of 720. In the second step, PyroNoise that removes homopolymer length error from 454 pyrosequencing was used for removal of base-call errors. In the third step, the primer and barcode sequences were removed and reverse-complement of some sequences was conducted. In the fourth step, human sequences were removed with BMTagger. In the fifth step, the chimera sequences were removed with UCHIME after Nearest Alignment Space Termination (NAST) alignment against the SILVA database. In the final trimming step, sequences less than 250 bases were removed. After completion of trimming, high-quality sequences were used for the bacterial community analysis.

#### Taxonomic assignment and taxonomic comparison

Taxonomic assignment of the high-quality reads was conducted using RDP classifier [14] which is a naïve Bayesian classifier and uses the RDP database. Among the taxonomically assigned reads, the reads with a confidence estimate of more than 0.8 were used for the taxonomic comparison. After taxonomic assignment of each read, the proportions of each bacterial group were calculated in each sample. The taxonomic comparisons between groups were conducted with the average relative abundance of each bacterial group in each sample group and standard error (SE) of the average relative abundance was calculated. For the clustering analysis, a heatmap was constructed with R script using the relative abundances of each bacterial class in the tissue samples of the patients and calculation of Euclidean distance and complete-linkage clustering was conducted. Wilcoxon signed-rank test in SPSS program (version 21, Inc., Chicago, IL) was used for the calculation of significant probability (*p*-value).

#### Diversity indices

Rarefaction, coverage, the total number of species observed in a sample (sobs), richness diversity indices such as abundance-based coverage estimators (ACE) and Chao estimator and even diversity indices such as Jackknife estimator, Shannon index, Inverse Simpson index were calculated with the unique sequences of each sample. For the calculation of diversity indices, unique sequences were selected by the mothur command ‘unique.seqs’ and distance matrix was generated with the mothur command ‘dist.seqs’ and clustered using phylip. Rarefaction was calculated with the mothur command ‘rarefaction.single’ and rarefaction curves were generated in Excel program. The coverage, sobs, and diversity indices were calculated with the mothur command ‘summary.single’. Wilcoxon signed-rank test was used for the calculation of *p*-value.

#### Principal coordinate analysis and UniFrac distance

For the principal coordinate analysis (PCoA), and calculation of UniFrac distance, representative sequences at the 3 % dissimilarity level were extracted from each samples and an environment file, which is containing the read information including the number of reads assigned to each representative sequences was generated. Distance matrix was constructed after NAST alignment against the SILVA database. The distance file was used for the tree generation using neighbor program [15] and the final PCoA results were generated with UniFrac. Program [16]. UniFrac distances were calculated with mothur command ‘unifrac unweighted’.

### Nucleotide sequence accession number

The DNA sequences from this metagenomic project have been deposited in the NCBI Short Read Archive under the bio-project number PRJNA240658.

## Results

### Patient characteristics and sequencing data

For the analysis of the intestinal microbial community, we collected 45 samples (10 feces and 35 mucosal tissues) from 35 CD patients and 30 samples (15 feces and 15 mucosal tissues) from 15 HC. Similar to the characteristics of CD patients reported in Korea, the CD group in the present study was male dominant with a mean age of 28.1 years. The mean period of disease duration was 54.9 months. Twenty-six patients had L3 lesions as defined by the Montreal classification [17] and 31.4 % of patients had peri-anal lesions. Fifteen patients were in active state, while 20 patients were in clinical and endoscopic remission state (Table 1).

**Table 1:** Clinical characteristics of enrolled subjects

	HC	CD
Number of subjects	15	35
Number of samples	30	45
Male:Female	14:1	26:9
Mean age (year)	35.0	28.1
BMI (kg/m^2^)	23.9	21.2
Mean disease duration (month)	–	54.9
Montreal classification^a^ (No. of patients)	–	L1B1 (2), L1B2 (2), L2B1 (4), L2B2 (1), L3B1 (17), L3B2 (5), L3B3 (4)
Perianal involvement (No. of patients)	–	11 (31.4 %)
Medication (No. of patients)	–	Prednisolone (5), 5-ASA (30), Azathioprine (22), Infliximab (9)

*HC* healthy control, *CD* Crohn’s disease, *BMI* body mass index, *5-ASA* 5-aminosalicylic acid. ^a^ Disease location and behavior are classified as L1-3 and B1-3, respectively; L1, ileal location; L2, colonic location; L3, ileocolonic location; B1, inflammatory behavior; B2, structuring behavior; B3, penetrating behavior

After read trimming, average 6341 and 6312 high quality reads were obtained from the fecal and mucosal tissue samples of HC, respectively (see Additional file 1: Table S1). From the CD patients, average 6698 and 11,363 high quality reads were obtained from the fecal and mucosal tissue samples, respectively.

### Global bacterial structure differed between feces and mucosal tissues in HC

**Fig. 1:**
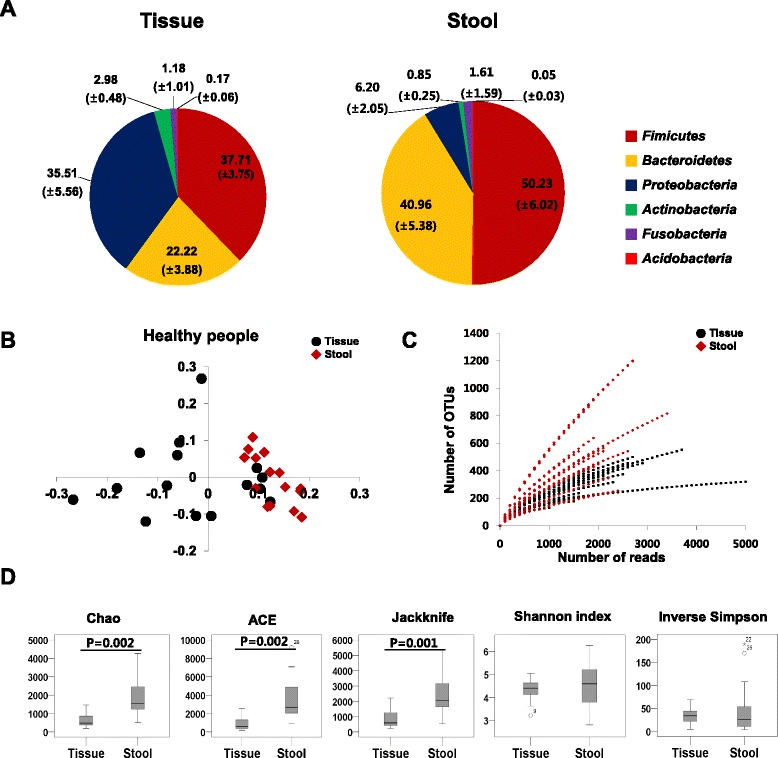
Intestinal microbial community structure in fecal and mucosal tissue samples of healthy control. **a** Taxonomic comparison at the phylum level. The numbers in parenthesis indicate the standard errors; **b** PCoA plot; **c** rarefaction curves; **d** diversity indices at the 3 % dissimilarity level. Chao and ACE indicate the richness diversity index. Jackknife, Shannon and Inverse Simpson indexes indicate the eveness diviersity index

We have compared the intestinal microbial community between fecal samples and mucosal tissue samples in HC. *Bacteroidetes* and *Firmicutes* were the major dominant bacterial phyla in the fecal samples of HC. On the contrary, the microbial community of the mucosal tissue samples of HC was mainly dominated with *Bacteroidetes*, *Firmicutes*, and *Proteobacteria* (Fig. 1a). Moreover, PCoA also showed the certain clustering pattern between the fecal and mucosal tissue samples in HC (Fig. 1b). The rarefaction curves and the values of the diversity indices between the fecal samples and tissue samples of HC indicated that the fecal microbiota have more richness values than the mucosal microbiota (Fig. 1c, d). However, there was no significant difference in the evenness diversity indices except for the Jackknife estimator.

### The intestinal microbial composition differed between CD and HC

**Fig. 2:**
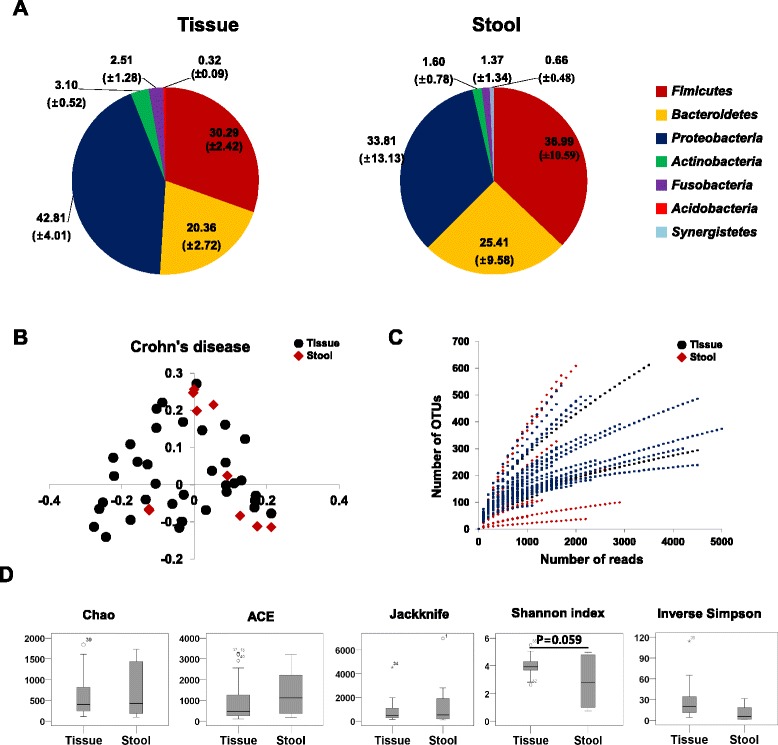
Intestinal microbial community structure in fecal and mucosal tissue samples of Crohn’s disease patients. **a** Taxonomic comparison at the phylum level. The numbers in parenthesis indicate the standard errors; **b** PCoA plot; **c** rarefaction curves; **d** diversity indices at the 3 % dissimilarity level. Chao and ACE indicate the richness diversity index. Jackknife, Shannon and Inverse Simpson indexes indicate the eveness diviersity index

At the phylum level, the relative abundance of *Proteobacteria* was increased in both fecal and mucosal tissues of CD patients compared to HC. Interestingly, the relative abundance of *Fusobacteria* was increased in the mucosal tissue samples of CD patients compared to HC, while the relative abundance of *Actinobacteria* was increased in the fecal samples of CD patients, although these differences were not statistically significant (Fig. 2a).

**Fig. 3:**
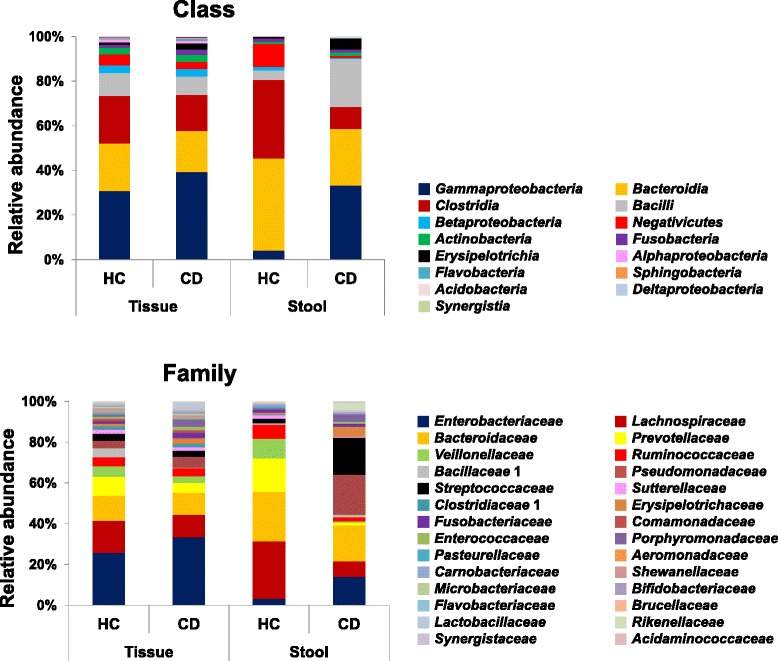
Taxonomic comparison of fecal and mucosal tissue samples between healthy control and Crohn’s disease patients at the class and family levels

At the class and family level, the relative abundances of *Enterobacteriaceae* of *Gammaproteobacteria* and *Fusobacteriaceae* of *Fusobacteria* were increased in mucosal tissue samples of CD patients compared to HC. Meawhile, in the fecal samples of CD patients, the relative abundances of *Enterobacteriaceae* and *Pseudomonadaceae* (*p* = 0.043) of *Gammaproteobacteria*, *Streptococcaceae* of *Bacilli*, and *Erysipelotrichaceae* of *Erysipelotrichia* were increased. These increase of relative abundances of *Gammaproteobacteria* and *Bacilli* in the fecal and mucosal tissues lead to concomitant decrease in the relative abundances of other classes and families such as *Bacteroidaceae* and *Prevotellaceae* (*p* = 0.028) of *Bacteroidia*, *Lachnospiraceae* (*p* = 0.028) and *Ruminococcaceae* (*p* = 0.028) of *Clostridia*, and *Veillonellaceae* (*p* = 0.017) of *Negativicutes* (Fig. 3, see Additional file 1: Table S2).

**Fig. 4:**
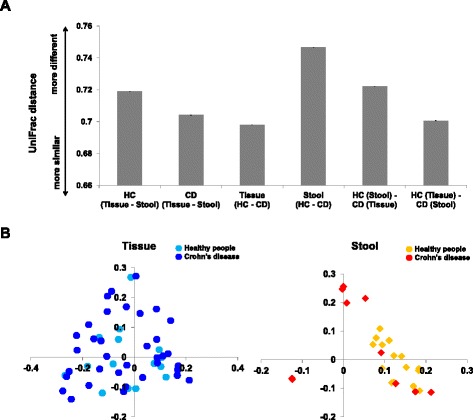
Unweighted UniFrac distance (**a**) and PCoA plot (**b**) between healthy control and Crohn’s disease patients

The diversity indices of fecal samples of CD patients were significantly decreased compared to those of HC, but there was no significant dfference of the diversity indices of the mucosal tissue samples between CD patients and HC (Figs. 1d and 2d). The taxonomic comparison also showed more significant differences of the fecal samples between CD and HC compared to mucosal tissue samples (Fig. 3, see Additional file 1: Table S2). Moreover, PCoA and UniFrac distance showed that similarity of the mucosal bacterial community between CD and HC was high, while similarity of the fecal bacterial community between two groups was very low (Fig. 4).

### Certain microbial population was related to clinical disease activity in CD patients

**Fig. 5:**
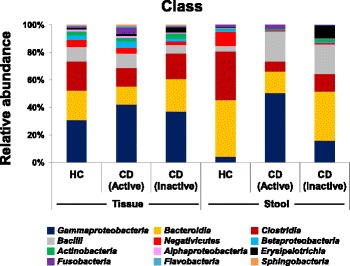
Taxonomic comparison of fecal and mucosal tissue samples between healthy control and Crohn’s disease patients at the class level according to the clinical disease activity

The relative abundances of *Gammaproteobacteria* and *Fusobacteria* were higher in both fecal and mucosal tissues of active-stage CD patients than CD patients without any active ulcer, although there was no significant difference (Fig. 5). At the genus level, the relative abundances of *Escherichia*/*Shigella* and *Pseudomonas* of *Gammaproteobacteria* and *Fusobacterium* of *Fusobacteria* were increased in both fecal and mucosal tissues of active CD patients. Interestingly, *Pseudomonas* was nearly absent in the fecal samples of HC, but its abundance was markedly increased in the fecal samples of active CD patients. On the other hand, the relative abundance of *Morganella*, which was only detected in the mucosal tissue samples, was increased in the active CD patients compared to CD patients with inactive stage. In the stool samples, the relative abundances of *Prevotella*, *Lachnospiraceae*, *Faecalibacterium*, and *Clostridium* XIVa were decreased in the active CD patients compared to HC (see Additional file 1: Table S3).

**Fig. 6:**
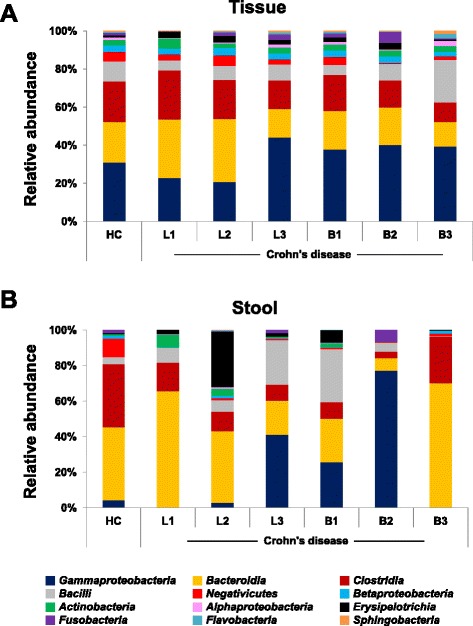
Taxonomic comparison of (**a**) mucosal tissues and (**b**) fecal samples between healthy control and Crohn’s disease patients at the class level according to the disease location and behavior

Analysis of the microbial community of CD patients at the class level according to disease location and behavior showed that the relative abundance of *Gammaproteobacteria* was highest in the fecal samples of patients with Montreal classification L3 and B2 lesions. *Fusobacteria* was also predominantly observed in both fecal and mucosal tissue samples of patients with L3 and B2 lesions (Fig. 6, see Additional file 1: Table S4).

### Therapeutic medications can affect the microbial population in CD patients

**Fig. 7:**
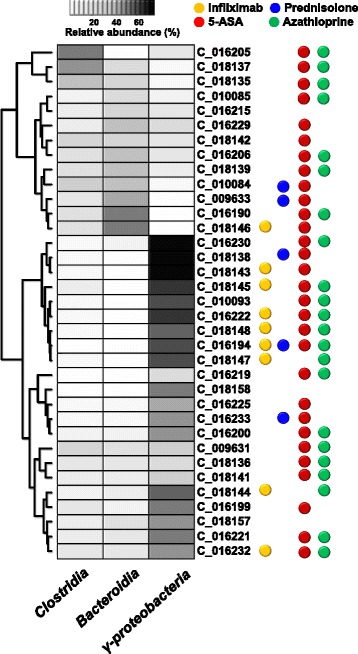
Construction of heatmap plot and clustering of the mucosal tissue samples of Crohn’s disease patients. The heatmap plot shows the relative abundance of the major three bacterial class of each patient. The dots colored by yellow, blue, red, and green indicate treatment of infliximab, prednisolone, 5-ASA, and azathioprine, respectively

Comparison of the difference on microbial community in CD patients according to the application of therapeutic medications revealed that a discrete clustering trend was seen between infliximab-treated group and infliximab-untreated group (Fig. 7). In addition, the relative abundance of *Gammaproteobacteria* in both fecal and tissue samples was increased in infliximab-treated group compared to infliximab-untreated group patients, while the relative abundance of *Fusobacteria* in both fecal and tissue samples was decreased in infliximab-treated group, although there was no significant difference (see Additional file 1: Table S5).

## Discussion

Our deep sequencing study showed that the intestinal microbial community in Korean CD patients differed from that of HC. The dysbiosis patterns in Korean CD patients, including decreased microbial diversity, increased relative abundances of *Proteobacteria* and *Fusobacteria* and decreased proportions of *Firmicutes* and *Bacteroidetes*, were similar to those of western CD patients. In addition, the intestinal microbial community structure of mucosal tissues samples differed from that of fecal samples. Finally, we demonstrated that the intestinal microbial community in CD patients was affected by clinical disease activity, disease location and behavior, and therapeutic medication such as anti-TNF agent.

Intestinal microbiota maintain the homeostasis of our body by keeping close contact with the host [18]. CD affects intestinal commensals in genetically susceptible hosts through various environmental factors, and excessive stimuli of the immune system ultimately leads to full-blown disease [19]. Compared with western population, Korean CD patients have distinct epidemiologic characteristics such as male predominance, frequent peri-anal lesions, and distinctive ileo-colonic manifestations, and the susceptibility genes commonly seen in western countries are rarely detected in Korean CD patients [9, 20]. However, since *NOD2* and *ATG16L1* mutations, which are closely related with bacterial handling, are observed in western CD population [5], and although not the same mutation, *ATG16L2* mutation related with regulation of bacteria-related signal was observed in Korean CD patients [9], it can be presumed that intestinal microbiota play an important role in the development of CD [21].

In the present study, Korean CD patients demonstrated alterations of intestinal microbial community structure differed from that of HC. The microbial diversity was decreased and the relative proportions of *Proteobacteria* and *Fusobacteria* were increased with concomitant decrease of relative abundances of *Firmicutes* and *Bacteroidetes* in CD patients compared to HC, especially in stool samples. These results are consistent with those of previous studies from western population [22, 23], suggesting an essential role of the intestinal microbial community in the development of CD throughout the world.

It has been well known that the relative proportions of *Proteobacteria* including adherent invasive *E. coli* and other numerous bacteria are increased in the mucosa of inflammatory bowel diseases (IBD) patients [24, 25]. In this study, *Gammaproteobacteria* class accounted for most of increase in the proportion of *Proteobacteria* phylum. And, *Escherichia/Shigella* genus were predominantly observed in both mucosal tissues and fecal samples of CD patients compared to HC, agreeing that *E. coli* strains play an important role in inducing chronic inflammation of intestinal tract.

On the other hand, the relative abundance of *Clostridia* class of *Firmicutes* phylum were decreased in both fecal and mucosal tissue samples of CD patients compared to HC. *Firmicutes* has been known to synthesize important short chain fatty acids, mainly acetate, propionate and butyrate, via fermentation of ingested dietary fiber within the intestine, which have profound effects on gut health as an energy source and inflammatory modulator [26]. Since intestinal microorganisms are closely related to food [11], it is interesting to know that decrease of relative proportion of *Firmicutes* was consistently observed in both Korean and western CD patients, even though the diet pattern of Korean patients is different from that of western counterpart. *Clostridia* class has also been reported to induce colonic regulatory T cells, which have a central role in the suppression of intestinal inflammation [27]. In addition, we showed that the relative proportions of *Faecalibacterium* and *Lachnospiraceae* were decreased in the fecal samples of CD patients. *Faecalibacterium prausnitzii*, an important protective commensal bacteria, has been known to be decreased in CD patients [28].

In the present study, we compared the intestinal microbial community between fecal samples and mucosal tissue samples in HC. In contrast to predominant proportions of *Firmicutes* and *Bacteroidetes* in fecal samples, the proportion of *Proteobacteria* was markedly increased in mucosal tissue samples with concomitant decrease of *Firmicutes* and *Bacteroidetes*. In addition, PCoA showed certain clustering pattern between fecal and mucosal tissue samples. Analysis of the microbial community between feces and mucosal tissues in CD patients also showed different bacterial community structures. These data are in accordance with previous studies demonstrating a considerable difference in composition between the mucosal and fecal samples [29, 30]. Considering mucosal associated bacterial profiles might have more important role in IBD than luminal bacteria, it is important to know that fecal microbial analysis does not fully reflect mucosal bacterial community structure [31].

It is well known that the intestinal microbial community of CD patients demonstrates dysbiosis different from healthy population [11, 12]. However, whether the changes of intestinal microbial composition in CD are primary or secondary events has not been determined yet. Transmission of colitis with fecal transplant from diseased donor to wildtype recipient, association of dysbiosis with genetic polymorphisms, and induction of immune mediated colitis through mono-association with commensal bacteria suggest dysbiosis as a primary cause of IBD. Meanwhile, similar dysbiotic patterns in nonspecific intestinal inflammation or infection, increased mucosal associated bacteria in patients with infectious colitis, and reversal of dysbiotic pattern with steroid treatment in IBD patients suggest secondary events of dysbiosis [32]. In our study, certain intestinal microbial population was related to clinical disease activity in CD patients. The relative abundances of *Gammaproteobacteria* and *Fusobacteria* were higher in both fecal and mucosal tissues of active-stage CD patients than inactive-stage CD patients. These results agree with several previous reports demonstrating disparities of intestinal microbial community between active versus quiescent IBD [33, 34]. We also demonstrated that the intestinal microbial composition in CD patients was related to disease location and behavior. Moreover, anti-TNF (infliximab) treatment affected a change in the intestinal microbial community structure in CD patients. These our results suggest dysbiosis as a secondary change of intestinal inflammation in CD.

This is the first study showing the unique change of intestinal microbiota community after infliximab treatment. 5-aminosalicylic acid (5-ASA), which is used for the treatment of IBD, activates peroxisome proliferator-activated receptor-r and affects luminal pH and intestinal mucosal pro-inflammatory cytokines. Although 5-ASA has previously been shown to alter the mucosal microbiota composition [29, 35], these effects of 5-ASA were not seen in our study because most of the enrolled patients had been taking 5-ASA. In contrast, a definitive clustering trend of microbial clades and differences of relative bacterial proportion were observed between infliximab-treated group and infliximab-untreated group. Interestingly, the relative abundance of *Gammaproteobacteria* was increased in both fecal and mucosal tissue samples of the infliximab-treated group. However, the differences of relative bacterial abundances between two groups were not significantly different. In addition, we did not investigate the sequential changes of intestinal microbial composition in same patients before and after infliximab treatment. Considering that the proportion of *Proteobacteria* increases as the severity of inflammation worsens, it is possible that the patients in the infliximab-treated group have more severe intestinal inflammation. Further studies are warranted to confirm potential influence of infliximab treatment on intestinal microbial community in CD patients.

In this study, the relative abundance of *Fusobacterium* genus was increased in both fecal and mucosal tissue samples of CD patients compared to HC. In addition, the proportion of *Fusobacteria* class were higher in CD patients with Montreal classification L3 lesion, which involves both small and large intestines. Moreover, the relative proportion of *Fusobacteria* class was decreased in the infliximab-treated group. *Fusobacteria* has recently become the bacterial strain of concern because of its relationship with IBD and colorectal cancer [36, 37]. *Fusobacterium nucleatum* is associated with chronic inflammation of the oral cavity, and it is commonly cultured from the intestinal mucosa of IBD patients. Because of its invasiveness, it is closely related with intestinal inflammation. Further studies using genetically susceptible mice monoassociated with *Fusobacteria* species will be needed to confirm whether this bacterial strain plays an important role in the development of chronic colitis as a pathobiont.

Our study faces several limitations. First of all, analysis at the species level could not be performed due to the technical limitations of metagenomic sequencing, and the extraordinary cost of whole genome sequencing. Secondly, most of our sequencing data regarding the differences of relative abundances of specific bacteria between groups could not reach statistically significant because of small sample size and relatively high variability of microbial composition in each group. Further study with a large population is required to confirm our data regarding such bacterial profiles of feces and mucosal tissues in Korean CD patients. Thirdly, in the present study, the effects of age on the intestinal microbial composition were minimized by enrolling Korean CD patients whose age was within the limits of CD prevalence and an age-matched HC group.

## Conclusions

Our study provides novel data that Korean CD patients demonstrate dysbiosis similar to western CD patients, who have different genetic and epidemiological backgrounds, implying that the intestinal microbial community may play an essential role in the development of CD. In addition, the intestinal microbial community structures are affected by clinical disease activity, disease location and anti-TNF treatment, suggesting dysbiosis as a secondary phenomenon of intestinal inflammation in CD. These results might provide a clue in understanding the pathogenesis of IBD in different genetic and epidemiologic backgrounds. Further comprehensive investigations involving the integration of genetic, microbiome, and immunologic aspects will hold a great promise for the prospect of personalized medical care in IBD field.

## Additional file

Additional file 1:
**Table S1.** Summary of Sequence Data of the Enrolled Samples. **Table S2.** Average Relative Abundance of Intestinal Microbiota at the Class and Family Levels. **Table S3.** Average Relative Abundance of Intestinal Microbiota at the Genus Level According to Clinical Disease Activity. **Table S4.** Taxonomic Comparison at the Class Level According to Disease Location and Behavior. **Table S5.** Taxonomic Comparison at the Class Level According to Treatment of Anti-TNF Agent. (DOCX 60 kb)
