# Supplementary material for: Does the intestinal microbial community of Korean Crohn’s disease patients differ from that of western patients?
Source: BMC Gastroenterol. 2016 Feb 29;16:28. doi: 10.1186/s12876-016-0437-0 (PMC4770608; doi:10.1186/s12876-016-0437-0)
Supplement: Additional file 1: — Table S1. Summary of Sequence Data of the Enrolled Samples. Table S2. Average Relative Abundance of Intestinal Microbiota at the Class and Family Levels. Table S3. Average Relative Abundance of Intestinal Microbiota at the Genus Level According to Clinical Disease Activity. Table S4. Taxonomic Comparison at the Class Level According to Disease Location and Behavior. Table S5. Taxonomic Comparison at the Class Level According to Treatment of Anti-TNF Agent. (DOCX 60 kb) [file 12876_2016_437_MOESM1_ESM.docx]

**Table S1.** Summary of Sequence Data of the Enrolled Samples

|  | **File name** | **Sample** | **HC/CD** | **No. of reads after trimming** | **No. of unique reads** | **Sobs^a^** | **Coverage^b^** |  | **File name** | **Sample** | **HC/CD** | **No. of reads after trimming** | **No. of unique reads** | **Sobs** | **Coverage** |
| --- | --- | --- | --- | --- | --- | --- | --- | --- | --- | --- | --- | --- | --- | --- | --- |
| 1 | C_016211 | T | HC | 7,432 | 2,995 | 463 | 0.91 | 41 | C_010084 | T | CD | 9,288 | 3,582 | 621 | 0.89 |
| 2 | C_016210 | S | HC | 5,046 | 1,998 | 441 | 0.85 | 42 | C_016196 | S | CD | 5,788 | 1,624 | 520 | 0.77 |
| 3 | C_016204 | T | HC | 5,262 | 2,161 | 235 | 0.96 | 43 | C_010093 | T | CD | 24,727 | 4,526 | 295 | 0.96 |
| 4 | C_016203 | S | HC | 9,253 | 2,645 | 548 | 0.87 | 44 | C_016195 | S | CD | 6,471 | 1,765 | 588 | 0.77 |
| 5 | C_016198 | T | HC | 16,628 | 5,034 | 321 | 0.98 | 45 | C_018147 | T | CD | 4,392 | 1,415 | 152 | 0.96 |
| 6 | C_016197 | S | HC | 9,237 | 2,292 | 245 | 0.93 | 46 | C_018152 | S | CD | 20,815 | 2,275 | 39 | 0.99 |
| 7 | C_018162 | T | HC | 3,033 | 1,484 | 273 | 0.93 | 47 | C_016232 | T | CD | 5,519 | 1,772 | 158 | 0.97 |
| 8 | C_018167 | S | HC | 5,139 | 2,140 | 571 | 0.82 | 48 | C_018149 | S | CD | 12,176 | 1,097 | 48 | 0.97 |
| 9 | C_016193 | T | HC | 7,190 | 1,597 | 180 | 0.98 | 49 | C_016219 | T | CD | 3,285 | 1,101 | 88 | 0.98 |
| 10 | C_016192 | S | HC | 8,230 | 2,257 | 351 | 0.89 | 50 | C_016215 | T | CD | 15,987 | 4,257 | 303 | 0.96 |
| 11 | C_018163 | T | HC | 8,087 | 1,985 | 228 | 0.94 | 51 | C_010085 | T | CD | 4,068 | 1,241 | 128 | 0.94 |
| 12 | C_018168 | S | HC | 4,964 | 2,188 | 556 | 0.83 | 52 | C_009633 | T | CD | 20,008 | 5,002 | 374 | 0.96 |
| 13 | C_018164 | T | HC | 5,807 | 2,580 | 380 | 0.93 | 53 | C_016205 | T | CD | 6,428 | 2,485 | 260 | 0.96 |
| 14 | C_018169 | S | HC | 4,361 | 1,982 | 658 | 0.75 | 54 | C_016230 | T | CD | 2,381 | 1,011 | 179 | 0.91 |
| 15 | C_018161 | T | HC | 4,811 | 2,357 | 318 | 0.94 | 55 | C_016229 | T | CD | 4,178 | 1,897 | 302 | 0.92 |
| 16 | C_018166 | S | HC | 3,411 | 1,827 | 873 | 0.64 | 56 | C_018138 | T | CD | 2,500 | 931 | 181 | 0.89 |
| 17 | C_016189 | T | HC | 4,331 | 2,179 | 369 | 0.92 | 57 | C_016225 | T | CD | 3,702 | 1,518 | 151 | 0.97 |
| 18 | C_016188 | S | HC | 6,194 | 2,078 | 460 | 0.85 | 58 | C_016222 | T | CD | 7,324 | 2,038 | 176 | 0.98 |
| 19 | C_018165 | T | HC | 2,671 | 1,080 | 134 | 0.95 | 59 | C_016221 | T | CD | 16,832 | 4,500 | 239 | 0.98 |
| 20 | C_018170 | S | HC | 4,591 | 2,718 | 1202 | 0.68 | 60 | C_018144 | T | CD | 5,354 | 1,864 | 192 | 0.96 |
| 21 | C_018172 | T | HC | 5,521 | 2,686 | 464 | 0.90 | 61 | C_018137 | T | CD | 4,523 | 1,765 | 548 | 0.80 |
| 22 | C_018176 | S | HC | 5,523 | 2,735 | 1213 | 0.66 | 62 | C_018135 | T | CD | 4,892 | 2,283 | 357 | 0.92 |
| 23 | C_018173 | T | HC | 5,800 | 2,769 | 507 | 0.89 | 63 | C_009631 | T | CD | 15,944 | 4,528 | 488 | 0.93 |
| 24 | C_018177 | S | HC | 3,738 | 1,093 | 191 | 0.88 | 64 | C_018146 | T | CD | 7,713 | 2,958 | 396 | 0.92 |
| 25 | C_018171 | T | HC | 9,756 | 3,771 | 558 | 0.91 | 65 | C_018148 | T | CD | 3,070 | 1,233 | 169 | 0.94 |
| 26 | C_018175 | S | HC | 12,373 | 3,462 | 826 | 0.83 | 66 | C_016206 | T | CD | 6,845 | 2,335 | 184 | 0.98 |
| 27 | C_018174 | T | HC | 4,148 | 1,672 | 242 | 0.93 | 67 | C_016200 | T | CD | 1,450 | 843 | 164 | 0.92 |
| 28 | C_018178 | S | HC | 3,160 | 1,620 | 546 | 0.76 | 68 | C_018157 | T | CD | 9,760 | 2,813 | 264 | 0.95 |
| 29 | C_016236 | T | HC | 4,198 | 1,560 | 187 | 0.96 | 69 | C_016199 | T | CD | 3,148 | 1,512 | 199 | 0.95 |
| 30 | C_016216 | S | HC | 9,899 | 2,424 | 256 | 0.94 | 70 | C_016233 | T | CD | 1,281 | 687 | 118 | 0.95 |
| 31 | C_018136 | T | CD | 3,788 | 1,511 | 236 | 0.93 | 71 | C_018141 | T | CD | 5,016 | 2,331 | 500 | 0.86 |
| 32 | C_018150 | S | CD | 5,999 | 1,685 | 338 | 0.87 | 72 | C_016194 | T | CD | 2,312 | 1,011 | 150 | 0.94 |
| 33 | C_018158 | T | CD | 5,755 | 1,914 | 162 | 0.97 | 73 | C_018142 | T | CD | 4,565 | 2,133 | 499 | 0.84 |
| 34 | C_018160 | S | CD | 7,462 | 1,304 | 108 | 0.96 | 74 | C_018159 | S | CD | 8,713 | 1,179 | 111 | 0.95 |
| 35 | C_018145 | T | CD | 7,741 | 2,375 | 217 | 0.96 | 75 | C_016190 | T | CD | 7,417 | 2,084 | 488 | 0.84 |
| 36 | C_016224 | S | CD | 29,093 | 2,936 | 101 | 0.98 |  |  |  |  |  |  |  |  |
| 37 | C_018139 | T | CD | 3,510 | 1,662 | 478 | 0.80 |  |  |  |  |  |  |  |  |
| 38 | C_018151 | S | CD | 5,818 | 2,056 | 621 | 0.79 |  |  |  |  |  |  |  |  |
| 39 | C_018143 | T | CD | 6,360 | 1,917 | 292 | 0.90 |  |  |  |  |  |  |  |  |
| 40 | C_016217 | S | CD | 11,291 | 2,659 | 226 | 0.95 |  |  |  |  |  |  |  |  |

^a^ sobs means the total number of species observed in a sample. ^b^ coverage means coverage at the 3% dissimilarity level. T, tissue; S, stool; HC, healthy controls; CD, Crohn’s disease.

**Table S2.** Average Relative Abundance of Intestinal Microbiota at the Class and Family Levels

|  | **Tissue** | | | | **Stool** | | | |
| --- | --- | --- | --- | --- | --- | --- | --- | --- |
|  | **HC** | | **CD** | | **HC** | | **CD** | |
|  | **Avg.^a^ (%)** | **SE^b^**  **(±)** | **Avg. (%)** | **SE**  **(±)** | **Avg. (%)** | **SE**  **(±)** | **Avg. (%)** | **SE**  **(±)** |
| **Class level** |  |  |  |  |  |  |  |  |
| ***Gammaproteobacteria*** | 30.78 | 5.45 | 38.06 | 3.96 | 4.23 | 1.79 | 33.20 | 13.29 |
| ***Bacteroidia*** | 21.33 | 3.90 | 19.07 | 2.82 | 41.09 | 5.38 | 25.42 | 9.59 |
| ***Clostridia*** | 21.22 | 3.19 | 16.80 | 2.15 | 35.31 | 5.38 | 9.72 | 3.33 |
| ***Bacilli*** | 10.30 | 3.58 | 7.91 | 1.92 | 4.12 | 2.08 | 21.60 | 12.16 |
| ***Betaproteobacteria*** | 3.29 | 0.50 | 3.35 | 0.47 | 1.69 | 0.76 | 0.46 | 0.22 |
| ***Negativicutes*** | 5.00 | 1.78 | 3.03 | 0.80 | 10.24 | 4.12 | 0.83 | 0.28 |
| ***Actinobacteria*** | 2.98 | 0.48 | 3.08 | 0.52 | 0.84 | 0.26 | 1.59 | 0.78 |
| ***Fusobacteria*** | 1.18 | 1.01 | 2.51 | 1.28 | 1.61 | 1.59 | 1.37 | 1.34 |
| ***Erysipelotrichia*** | 1.21 | 0.24 | 2.53 | 0.53 | 0.65 | 0.16 | 4.81 | 3.16 |
| ***Alphaproteobacteria*** | 1.28 | 0.35 | 1.27 | 0.26 | 0.06 | 0.02 | 0.11 | 0.08 |
| ***Flavobacteria*** | 0.56 | 0.18 | 0.84 | 0.23 | 0.01 | 0.01 | 0.06 | 0.06 |
| ***Sphingobacteria*** | 0.44 | 0.13 | 0.57 | 0.18 | 0.02 | 0.01 | 0.01 | 0.01 |
| ***Acidobacteria*** | 0.16 | 0.06 | 0.31 | 0.09 | 0.05 | 0.03 | 0.00 | 0.00 |
| ***Deltaproteobacteria*** | 0.10 | 0.05 | 0.17 | 0.06 | 0.03 | 0.01 | 0.01 | 0.01 |
| ***Synergistia*** | 0.01 | 0.01 | 0.06 | 0.04 | 0.00 | 0.00 | 0.65 | 0.47 |
| **Family level** |  |  |  |  |  |  |  |  |
| ***Enterobacteriaceae*** | 24.22 | 4.97 | 30.34 | 3.57 | 3.26 | 1.69 | 13.77 | 8.08 |
| ***Lachnospiraceae*** | 14.60 | 2.89 | 11.01 | 1.52 | 27.78 | 4.55 | 7.48 | 2.54 |
| ***Bacteroidaceae*** | 11.53 | 2.92 | 10.35 | 2.26 | 23.97 | 5.27 | 17.39 | 8.31 |
| ***Prevotellaceae*** | 8.64 | 3.73 | 5.13 | 1.83 | 16.09 | 7.03 | 1.17 | 0.86 |
| ***Veillonellaceae*** | 4.79 | 1.81 | 2.87 | 0.77 | 9.63 | 4.08 | 0.73 | 0.28 |
| ***Ruminococcaceae*** | 4.33 | 1.14 | 3.80 | 0.92 | 6.81 | 1.41 | 2.03 | 1.04 |
| ***Bacillaceae* 1** | 4.02 | 2.85 | 0.45 | 0.45 | 0.53 | 0.41 | 1.13 | 0.50 |
| ***Pseudomonadaceae*** | 3.61 | 0.67 | 5.22 | 1.32 | 0.00 | 0.00 | 19.35 | 12.88 |
| ***Streptococcaceae*** | 3.21 | 1.08 | 2.20 | 0.49 | 2.26 | 2.03 | 17.86 | 11.75 |
| ***Sutterellaceae*** | 1.69 | 0.49 | 1.62 | 0.48 | 1.64 | 0.76 | 0.44 | 0.21 |
| ***Clostridiaceae* 1** | 1.62 | 0.34 | 1.67 | 0.28 | 0.56 | 0.45 | 0.06 | 0.03 |
| ***Erysipelotrichaceae*** | 1.21 | 0.24 | 2.54 | 0.53 | 0.62 | 0.15 | 4.80 | 3.16 |
| ***Fusobacteriaceae*** | 1.17 | 1.02 | 2.50 | 1.28 | 1.61 | 1.59 | 1.37 | 1.35 |
| ***Comamonadaceae*** | 1.07 | 0.26 | 1.15 | 0.25 | 0.00 | 0.00 | 0.01 | 0.01 |
| ***Enterococcaceae*** | 1.01 | 0.22 | 1.42 | 0.53 | 0.03 | 0.02 | 1.11 | 0.69 |
| ***Porphyromonadaceae*** | 0.91 | 0.18 | 3.29 | 0.89 | 0.71 | 0.24 | 3.49 | 2.70 |
| ***Pasteurellaceae*** | 0.87 | 0.21 | 0.60 | 0.14 | 0.92 | 0.64 | 0.02 | 0.02 |
| ***Aeromonadaceae*** | 0.80 | 0.21 | 0.79 | 0.14 | 0.00 | 0.00 | 0.00 | 0.00 |
| ***Carnobacteriaceae*** | 0.70 | 0.23 | 0.27 | 0.07 | 0.40 | 0.37 | 0.03 | 0.02 |
| ***Shewanellaceae*** | 0.62 | 0.16 | 0.59 | 0.11 | 0.00 | 0.00 | 0.00 | 0.00 |
| ***Microbacteriaceae*** | 0.62 | 0.17 | 0.62 | 0.12 | 0.00 | 0.00 | 0.00 | 0.00 |
| ***Bifidobacteriaceae*** | 0.56 | 0.22 | 1.08 | 0.47 | 0.45 | 0.17 | 0.87 | 0.46 |
| ***Flavobacteriaceae*** | 0.55 | 0.18 | 0.83 | 0.23 | 0.01 | 0.01 | 0.06 | 0.06 |
| ***Brucellaceae*** | 0.51 | 0.13 | 0.56 | 0.10 | 0.00 | 0.00 | 0.00 | 0.00 |
| ***Lactobacillaceae*** | 0.37 | 0.17 | 2.94 | 1.72 | 0.37 | 0.16 | 1.03 | 0.58 |
| ***Acidaminococcaceae*** | 0.22 | 0.12 | 0.17 | 0.09 | 0.60 | 0.50 | 0.10 | 0.10 |
| ***Rikenellaceae*** | 0.18 | 0.08 | 0.34 | 0.11 | 0.38 | 0.12 | 3.41 | 3.37 |
| ***Synergistaceae*** | 0.01 | 0.01 | 0.06 | 0.04 | 0.00 | 0.00 | 0.65 | 0.48 |

^a^ Avg. (%) means the average of the relative abundances. ^b^ SE (±) means the standard error for the average values. HC, healthy control; CD, Crohn’s disease.

**Table S3.** Average Relative Abundance of Intestinal Microbiota at the Genus Level According to Clinical Disease Activity

|  | **Tissue** | | | | | | | **Stool** | | | | | | | |
| --- | --- | --- | --- | --- | --- | --- | --- | --- | --- | --- | --- | --- | --- | --- | --- |
|  | **HC** | | **CD** | | | | | **HC** | | | **CD** | | | | |
| **Status** |  | | | **Active** | | **Inactive** | | |  | | | **Active** | | **Inactive** | |
| **Genus** | **Avg.**^a^ **(%)** | **SE**^b^  **(±)** | | **Avg. (%)** | **SE**  **(±)** | **Avg. (%)** | **SE**  **(±)** | | **Avg. (%)** | **SE**  **(±)** | | **Avg. (%)** | **SE**  **(±)** | **Avg. (%)** | **SE**  **(±)** |
| ***Bacteroides*** | 11.60 | 2.93 | | 10.19 | 2.14 | 11.86 | 3.10 | | 24.21 | 5.32 | | 15.68 | 12.49 | 19.28 | 12.47 |
| ***Escherichia*/*Shigella*** | 9.42 | 4.98 | | 16.08 | 3.20 | 16.78 | 4.94 | | 3.03 | 1.67 | | 11.44 | 11.42 | 15.62 | 12.82 |
| ***Morganella*** | 8.77 | 2.31 | | 7.95 | 1.48 | 5.46 | 1.24 | | 0.00 | 0.00 | | 0.00 | 0.00 | 0.00 | 0.00 |
| ***Prevotella*** | 8.29 | 3.68 | | 4.38 | 1.57 | 7.01 | 2.60 | | 14.84 | 7.10 | | 0.00 | 0.00 | 1.69 | 1.67 |
| ***Lachnospiraceae* incertae sedis** | 6.87 | 1.37 | | 4.06 | 0.57 | 4.82 | 0.72 | | 15.41 | 2.52 | | 3.65 | 2.44 | 4.33 | 2.09 |
| ***Bacillus*** | 3.98 | 2.83 | | 0.41 | 0.41 | 0.78 | 0.77 | | 0.51 | 0.40 | | 0.53 | 0.53 | 1.70 | 0.80 |
| ***Pseudomonas*** | 3.64 | 0.68 | | 5.15 | 1.24 | 5.02 | 2.14 | | 0.00 | 0.00 | | 38.66 | 23.66 | 0.04 | 0.03 |
| ***Faecalibacterium*** | 3.45 | 0.92 | | 2.64 | 0.66 | 2.94 | 0.89 | | 5.21 | 1.22 | | 1.67 | 1.67 | 1.72 | 1.02 |
| ***Streptococcus*** | 3.23 | 1.10 | | 2.66 | 0.74 | 2.95 | 0.81 | | 2.21 | 2.05 | | 19.11 | 19.09 | 16.83 | 16.22 |
| ***Dorea*** | 2.49 | 0.79 | | 1.30 | 0.38 | 2.18 | 0.66 | | 2.62 | 0.65 | | 0.25 | 0.25 | 0.77 | 0.39 |
| ***Clostridium* XlVa** | 2.10 | 0.58 | | 2.29 | 0.47 | 2.48 | 0.72 | | 3.20 | 0.79 | | 0.52 | 0.32 | 2.15 | 1.22 |
| ***Providencia*** | 2.10 | 0.54 | | 2.05 | 0.38 | 1.41 | 0.32 | | 0.00 | 0.00 | | 0.00 | 0.00 | 0.00 | 0.00 |
| ***Megamonas*** | 1.60 | 1.38 | | 1.11 | 0.63 | 0.70 | 0.59 | | 5.72 | 3.62 | | 0.26 | 0.26 | 0.00 | 0.00 |
| ***Clostridium* sensu stricto** | 1.59 | 0.33 | | 1.61 | 0.26 | 1.30 | 0.27 | | 0.54 | 0.43 | | 0.01 | 0.01 | 0.11 | 0.05 |
| ***Veillonella*** | 1.38 | 0.82 | | 0.99 | 0.30 | 0.67 | 0.15 | | 0.84 | 0.72 | | 0.08 | 0.08 | 0.53 | 0.51 |
| ***Sutterella*** | 1.31 | 0.44 | | 1.04 | 0.30 | 0.77 | 0.30 | | 1.27 | 0.72 | | 0.02 | 0.02 | 0.62 | 0.36 |
| ***Clostridium* XIX** | 1.05 | 1.03 | | 1.77 | 1.17 | 0.36 | 0.36 | | 1.59 | 1.59 | | 0.01 | 0.01 | 0.00 | 0.00 |
| ***Blautia*** | 1.03 | 0.44 | | 0.84 | 0.19 | 1.15 | 0.28 | | 0.75 | 0.24 | | 0.44 | 0.44 | 0.78 | 0.52 |
| ***Proteus*** | 0.98 | 0.24 | | 0.94 | 0.17 | 0.70 | 0.14 | | 0.00 | 0.00 | | 0.00 | 0.00 | 0.00 | 0.00 |
| ***Comamonas*** | 0.96 | 0.25 | | 0.97 | 0.21 | 0.64 | 0.16 | | 0.00 | 0.00 | | 0.00 | 0.00 | 0.00 | 0.00 |
| ***Vagococcus*** | 0.89 | 0.22 | | 0.83 | 0.14 | 0.66 | 0.13 | | 0.00 | 0.00 | | 0.00 | 0.00 | 0.00 | 0.00 |
| ***Enterobacter*** | 0.84 | 0.21 | | 1.32 | 0.34 | 1.32 | 0.56 | | 0.03 | 0.02 | | 0.02 | 0.01 | 0.05 | 0.03 |
| ***Aeromonas*** | 0.81 | 0.21 | | 0.78 | 0.14 | 0.58 | 0.12 | | 0.00 | 0.00 | | 0.00 | 0.00 | 0.00 | 0.00 |
| ***Roseburia*** | 0.72 | 0.17 | | 0.46 | 0.15 | 0.66 | 0.24 | | 3.01 | 1.10 | | 0.12 | 0.11 | 0.63 | 0.60 |
| ***Serratia*** | 0.64 | 0.21 | | 0.89 | 0.24 | 0.68 | 0.34 | | 0.00 | 0.00 | | 0.00 | 0.00 | 0.00 | 0.00 |
| ***Bifidobacterium*** | 0.57 | 0.22 | | 1.03 | 0.43 | 1.57 | 0.80 | | 0.46 | 0.17 | | 0.84 | 0.83 | 0.92 | 0.52 |
| ***Dialister*** | 0.54 | 0.25 | | 0.72 | 0.28 | 0.83 | 0.45 | | 1.71 | 0.76 | | 0.34 | 0.34 | 0.24 | 0.15 |
| ***Erysipelotrichaceae* incertae sedis** | 0.49 | 0.20 | | 0.51 | 0.28 | 0.90 | 0.52 | | 0.26 | 0.10 | | 0.00 | 0.00 | 6.60 | 6.04 |
| ***Megasphaera*** | 0.48 | 0.28 | | 0.07 | 0.03 | 0.11 | 0.04 | | 1.01 | 0.72 | | 0.00 | 0.00 | 0.02 | 0.01 |
| ***Parabacteroides*** | 0.44 | 0.13 | | 1.67 | 0.51 | 1.55 | 0.63 | | 0.38 | 0.09 | | 0.09 | 0.09 | 1.58 | 1.45 |
| ***Clostridium* XVIII** | 0.43 | 0.10 | | 1.32 | 0.43 | 2.13 | 0.76 | | 0.28 | 0.12 | | 0.10 | 0.06 | 2.91 | 2.70 |
| ***Parasutterella*** | 0.38 | 0.30 | | 0.54 | 0.37 | 0.05 | 0.02 | | 0.38 | 0.35 | | 0.25 | 0.25 | 0.00 | 0.00 |
| ***Syntrophococcus*** | 0.37 | 0.09 | | 0.27 | 0.10 | 0.47 | 0.17 | | 1.54 | 0.67 | | 0.00 | 0.00 | 0.52 | 0.48 |
| ***Lactobacillus*** | 0.36 | 0.17 | | 2.70 | 1.59 | 0.65 | 0.39 | | 0.38 | 0.17 | | 0.70 | 0.68 | 0.10 | 0.07 |
| ***Alistipes*** | 0.18 | 0.08 | | 0.31 | 0.10 | 0.29 | 0.13 | | 0.39 | 0.13 | | 0.01 | 0.01 | 6.94 | 6.87 |
| ***Fusobacterium*** | 0.12 | 0.04 | | 0.87 | 0.34 | 0.38 | 0.21 | | 0.02 | 0.01 | | 2.70 | 2.68 | 0.03 | 0.02 |
| ***Enterococcus*** | 0.10 | 0.07 | | 0.71 | 0.48 | 0.12 | 0.10 | | 0.03 | 0.02 | | 0.08 | 0.07 | 0.82 | 0.68 |
| ***Porphyromonas*** | 0.05 | 0.03 | | 0.77 | 0.62 | 1.46 | 1.18 | | 0.00 | 0.00 | | 0.00 | 0.00 | 5.30 | 5.30 |
| ***Tetragenococcus*** | 0.03 | 0.01 | | 0.00 | 0.00 | 0.00 | 0.00 | | 0.00 | 0.00 | | 1.31 | 1.31 | 0.00 | 0.00 |
| ***Citrobacter*** | 0.02 | 0.01 | | 0.91 | 0.81 | 1.70 | 1.54 | | 0.00 | 0.00 | | 0.01 | 0.01 | 0.00 | 0.00 |

^a^ Avg. (%) means the average of the relative abundances. ^b^ SE (±) means the standard error for the average values. HC, healthy control; CD, Crohn’s disease.

**Table S4.** Taxonomic Comparison at the Class Level According to Disease Location and Behavior

|  | **HC** | | **CD** | | | | | | | | | | | |
| --- | --- | --- | --- | --- | --- | --- | --- | --- | --- | --- | --- | --- | --- | --- |
|  |  |  | **L1** | | **L2** | | **L3** | | **B1** | | **B2** | | **B3** | |
| **Tissue** | **Avg.**^a^ **(%)** | **SE**^b^ **(±)** | **Avg. (%)** | **SE (±)** | **Avg. (%)** | **SE (±)** | **Avg. (%)** | **SE (±)** | **Avg. (%)** | **SE (±)** | **Avg. (%)** | **SE (±)** | **Avg. (%)** | **SE (±)** |
| ***Gammaproteobacteria*** | 30.78 | 5.45 | 22.46 | 11.76 | 20.59 | 10.11 | 43.82 | 4.23 | 37.43 | 5.04 | 39.76 | 9.45 | 38.88 | 8.79 |
| ***Bacteroidia*** | 21.33 | 3.9 | 29.98 | 7.58 | 32.59 | 8.73 | 14.79 | 2.88 | 19.94 | 3.37 | 19.63 | 7.01 | 12.84 | 8.86 |
| ***Clostridia*** | 21.22 | 3.19 | 25.36 | 7.62 | 20.36 | 6.35 | 14.8 | 2.34 | 18.71 | 2.74 | 14.01 | 4.32 | 10.22 | 4.77 |
| ***Bacilli*** | 10.3 | 3.58 | 4.97 | 3.33 | 7.14 | 3.89 | 8.51 | 2.45 | 5.4 | 1.23 | 8.51 | 3.7 | 21.93 | 13.04 |
| ***Negativicutes*** | 5 | 1.78 | 3.3 | 2 | 5.71 | 2.94 | 2.48 | 0.88 | 3.97 | 1.12 | 0.58 | 0.28 | 1.69 | 0.81 |
| ***Betaproteobacteria*** | 3.29 | 0.5 | 3.01 | 1.26 | 3.74 | 1.3 | 3.33 | 0.56 | 3.54 | 0.63 | 3.25 | 0.88 | 2.4 | 0.74 |
| ***Actinobacteria*** | 2.98 | 0.48 | 5.08 | 2.7 | 2.25 | 0.68 | 2.93 | 0.55 | 3 | 0.59 | 3.32 | 1.6 | 3.09 | 1.05 |
| ***Alphaproteobacteria*** | 1.28 | 0.35 | 0.42 | 0.3 | 0.61 | 0.36 | 1.53 | 0.33 | 1.27 | 0.35 | 0.49 | 0.31 | 2.6 | 0.23 |
| ***Erysipelotrichia*** | 1.21 | 0.24 | 3.1 | 1.11 | 3.36 | 1.86 | 2.29 | 0.61 | 2.57 | 0.63 | 3.34 | 1.51 | 0.87 | 0.34 |
| ***Fusobacteria*** | 1.18 | 1.01 | 0.03 | 0.01 | 1.93 | 1.8 | 3.01 | 1.68 | 1.88 | 0.9 | 5.84 | 5.76 | 0.51 | 0.38 |
| ***Flavobacteria*** | 0.56 | 0.18 | 0.13 | 0.11 | 0.34 | 0.16 | 1.05 | 0.3 | 0.77 | 0.26 | 0.21 | 0.14 | 2.42 | 1.05 |
| ***Sphingobacteria*** | 0.44 | 0.13 | 0.14 | 0.12 | 0.28 | 0.14 | 0.7 | 0.23 | 0.55 | 0.23 | 0.15 | 0.09 | 1.45 | 0.58 |
|  | **HC** | | **CD** | | | | | | | | | | | |
|  |  |  | **L1** | | **L2** | | **L3** | | **B1** | | **B2** | | **B3** | |
| **Stool** | **Avg. (%)** | **SE (±)** | **Avg. (%)** | **SE (±)** | **Avg. (%)** | **SE (±)** | **Avg. (%)** | **SE (±)** | **Avg. (%)** | **SE (±)** | **Avg. (%)** | **SE (±)** | **Avg. (%)** | **SE (±)** |
| ***Gammaproteobacteria*** | 4.23 | 1.79 | 0.00 | - | 2.64 | - | 41.17 | 15.44 | 25.39 | 15.20 | 77.13 | 19.93 | 0.00 | - |
| ***Bacteroidia*** | 41.09 | 5.38 | 62.15 | - | 39.44 | - | 19.07 | 10.70 | 24.29 | 11.43 | 6.96 | 6.96 | 70.19 | - |
| ***Clostridia*** | 35.31 | 5.38 | 15.17 | - | 10.67 | - | 8.92 | 4.14 | 9.06 | 3.85 | 3.77 | 3.77 | 26.20 | - |
| ***Bacilli*** | 4.12 | 2.08 | 7.80 | - | 6.43 | - | 25.22 | 15.11 | 29.38 | 16.81 | 4.99 | 2.27 | 0.32 | - |
| ***Negativicutes*** | 10.24 | 4.12 | 0.13 | - | 0.89 | - | 0.92 | 0.34 | 0.95 | 0.38 | 0.20 | 0.20 | 1.27 | - |
| ***Betaproteobacteria*** | 1.69 | 0.76 | 0.10 | - | 1.51 | - | 0.37 | 0.23 | 0.42 | 0.25 | 0.04 | 0.04 | 1.58 | - |
| ***Actinobacteria*** | 0.84 | 0.26 | 6.81 | - | 3.89 | - | 0.65 | 0.51 | 2.24 | 1.03 | 0.00 | 0.00 | 0.23 | - |
| ***Alphaproteobacteria*** | 0.06 | 0.02 | 0.18 | - | 0.85 | - | 0.01 | 0.01 | 0.16 | 0.12 | 0.00 | 0.00 | 0.00 | - |
| ***Erysipelotrichia*** | 0.65 | 0.16 | 2.22 | - | 30.49 | - | 1.92 | 1.71 | 6.82 | 4.37 | 0.06 | 0.06 | 0.19 | - |
| ***Fusobacteria*** | 1.61 | 1.59 | 0.00 | - | 0.07 | - | 1.71 | 1.68 | 0.03 | 0.01 | 6.74 | 6.74 | 0.00 | - |
| ***Flavobacteria*** | 0.01 | 0.01 | 0.00 | - | 0.59 | - | 0.00 | 0.00 | 0.09 | 0.08 | 0.00 | 0.00 | 0.00 | - |
| ***Sphingobacteria*** | 0.02 | 0.01 | 0.00 | - | 0.09 | - | 0.00 | 0.00 | 0.01 | 0.01 | 0.00 | 0.00 | 0.00 | - |

^a^ Avg. (%) means the average of the relative abundances. ^b^ SE (±) means the standard error for the average values. HC, healthy control; CD, Crohn’s disease. '-' means that the lesion included only one sample, so standard error was not calculated. Disease location and behavior are classified as L1-3 and B1-3, respectively; L1, ileal location; L2, colonic location; L3, ileocolonic location; B1, inflammatory behavior; B2, structuring behavior; B3, penetrating behavior.

**Table S5.** Taxonomic Comparison at the Class Level According to Treatment of Anti-TNF Agent

|  | **Tissue** | | | | | | **Stool** | | | | | |
| --- | --- | --- | --- | --- | --- | --- | --- | --- | --- | --- | --- | --- |
|  | **HC** | | **CD** | | | | **HC** | | **CD** | | | |
|  |  | | Infliximab treated | | Infliximab untreated | |  | | Infliximab treated | | Infliximab untreated | |
|  | **Avg.**^a^ **(%)** | **SE**^b^ **(±)** | **Avg. (%)** | **SE (±)** | **Avg. (%)** | **SE (±)** | **Avg. (%)** | **SE (±)** | **Avg. (%)** | **SE (±)** | **Avg. (%)** | **SE (±)** |
| ***Gammaproteobacteria*** | 30.78 | 5.45 | 54.97 | 6.80 | 32.20 | 4.28 | 4.23 | 1.79 | 81.78 | 15.28 | 21.05 | 13.04 |
| ***Bacteroidia*** | 21.33 | 3.90 | 10.89 | 5.83 | 21.90 | 3.09 | 41.09 | 5.38 | 1.83 | 1.83 | 31.31 | 11.09 |
| ***Clostridia*** | 21.22 | 3.19 | 10.30 | 2.27 | 19.05 | 2.67 | 35.31 | 5.38 | 4.83 | 4.83 | 10.94 | 3.98 |
| ***Bacilli*** | 10.30 | 3.58 | 10.67 | 2.68 | 6.95 | 2.41 | 4.12 | 2.08 | 2.84 | 0.12 | 26.29 | 14.90 |
| ***Negativicutes*** | 5.00 | 1.78 | 2.49 | 1.33 | 3.22 | 0.99 | 10.24 | 4.12 | 1.32 | 1.32 | 0.71 | 0.23 |
| ***Betaproteobacteria*** | 3.29 | 0.50 | 2.88 | 0.67 | 3.52 | 0.59 | 1.69 | 0.76 | 0.01 | 0.01 | 0.57 | 0.26 |
| ***Actinobacteria*** | 2.98 | 0.48 | 2.73 | 0.71 | 3.20 | 0.66 | 0.84 | 0.26 | 0.24 | 0.24 | 1.93 | 0.94 |
| ***Alphaproteobacteria*** | 1.28 | 0.35 | 1.59 | 0.52 | 1.16 | 0.31 | 0.06 | 0.02 | 0.00 | 0.00 | 0.14 | 0.10 |
| ***Erysipelotrichia*** | 1.21 | 0.24 | 1.36 | 0.87 | 2.94 | 0.63 | 0.65 | 0.16 | 6.93 | 6.93 | 4.28 | 3.75 |
| ***Fusobacteria*** | 1.18 | 1.01 | 0.33 | 0.29 | 3.27 | 1.70 | 1.61 | 1.59 | 0.04 | 0.04 | 1.71 | 1.68 |
| ***Flavobacteria*** | 0.56 | 0.18 | 0.53 | 0.21 | 0.95 | 0.30 | 0.01 | 0.01 | 0.00 | 0.00 | 0.08 | 0.07 |
| ***Sphingobacteria*** | 0.44 | 0.13 | 0.43 | 0.15 | 0.62 | 0.23 | 0.02 | 0.01 | 0.00 | 0.00 | 0.01 | 0.01 |

^a^ Avg. (%) means the average of the relative abundances. ^b^ SE (±) means the standard error for the average values. HC, healthy control; CD, Crohn’s disease.
